# Glutamine: An Obligatory Parenteral Nutrition Substrate in Critical Care Therapy

**DOI:** 10.1155/2015/545467

**Published:** 2015-10-01

**Authors:** Peter Stehle, Katharina S. Kuhn

**Affiliations:** Department of Nutrition and Food Sciences, University of Bonn, 53115 Bonn, Germany

## Abstract

Critical illness is characterized by glutamine depletion owing to increased metabolic demand. Glutamine is essential to maintain intestinal integrity and function, sustain immunologic response, and maintain antioxidative balance. Insufficient endogenous availability of glutamine may impair outcome in critically ill patients. Consequently, glutamine has been considered to be a conditionally essential amino acid and a necessary component to complete any parenteral nutrition regimen. Recently, this scientifically sound recommendation has been questioned, primarily based on controversial findings from a large multicentre study published in 2013 that evoked considerable uncertainty among clinicians. The present review was conceived to clarify the most important questions surrounding glutamine supplementation in critical care. This was achieved by addressing the role of glutamine in the pathophysiology of critical illness, summarizing recent clinical studies in patients receiving parenteral nutrition with intravenous glutamine, and describing practical concepts for providing parenteral glutamine in critical care.

## 1. Introduction

The amino acid glutamine (Gln) plays a central role in human nitrogen, protein, and energy metabolism. Gln transports nitrogen between cells and/or organs and serves as a metabolic fuel—in addition to glucose or as an alternative—in rapidly proliferating cells. Gln is a precursor for protein, nucleotide, and nucleic acid synthesis and also regulates cellular pathways and related functions [1–3]. Gln is the most abundant extracellular and intracellular free amino acid in the human body [1]. Blood Gln concentration reflects the short-term balance between exogenous supply, endogenous release, and consumption by organs and cells (e.g., enterocytes and immunocompetent cells). Intracellular concentrations in muscle tissue, representing more than 50% of the body Gln pool [4], can be seen as a long-term biomarker to evaluate Gln status [5].

Under physiological conditions, sufficient endogenous Gln stores (as judged by normal intracellular concentrations) are maintained by both daily nutritional intake (80 g of mixed protein contains approximately 10 g Gln) and by endogenous synthesis which occurs mainly in skeletal muscle and the liver [6]. However, during situations characterized by increased release of catabolic hormones and/or inflammatory cytokines, changes in amino acid and protein metabolism lead to increased risk of a negative Gln balance leading to whole body metabolic derangement and clinical symptoms [7–9]. This pathophysiological situation has to be considered when adequate nutritional support is designed for patients who are not able to consume food, especially protein, in sufficient amounts, such as in critically ill patients admitted to the intensive care unit (ICU).

Routine Gln supplementation has long been recommended for critically ill patients receiving parenteral nutrition (PN) [10, 11]. Numerous clinical trials and meta-analyses indicate that counteracting Gln deficiency helps to normalize metabolism and, most importantly, improves outcome [12, 13]. Recently, this scientifically sound recommendation has been questioned, [14, 15] primarily based on the controversial findings from a large multicentre study published in 2013 [16–18] which caused considerable uncertainty amongst clinicians. The present review was conceived to clarify these important questions surrounding Gln supplementation in critical care. This will be achieved by addressing the role of Gln in the pathophysiology of critical illness, summarizing recent clinical studies in patients receiving PN with intravenous Gln, and describing practical concepts for providing parenteral Gln in critical care.

## 2. Gln: An Indispensable Nutrient in Critical Care

The first reports of muscle Gln depletion in trauma and critical illness emerged in the late 1970s and early 1980s [19–21]. Thereafter, it has been demonstrated repeatedly that critical illnesses such as trauma [22] and/or sepsis [23–25], burns [26, 27], and severe acute pancreatitis (AP) [28] are associated with profound intracellular Gln depletion. This can also be reflected in decreased plasma Gln levels as seen in mixed [29], medical [30], or surgical [31] ICU patients, trauma [22, 32, 33], sepsis [34, 35], burns [36, 37], and severe AP [28]. A decrease of free Gln in muscle to approximately 50% of normal levels has long been considered a hallmark of the metabolic responses to stress [38].

Triggered by endocrine mechanisms, Gln-consuming organs, like the gut, gut-associated lymphoid tissue (GALT), and the kidneys, all enhance the uptake of Gln to support the synthesis of acute-phase proteins (e.g., heat shock proteins, HSP) [39, 40], optimize the cellular immune response [41, 42], increase antioxidative defence (e.g., synthesis of glutathione) [1, 43], and maintain cellular structures (e.g., barrier functions of the gastrointestinal tract) [40, 44, 45]. As a consequence of this drastically increased metabolic demand for Gln, endogenous Gln production cannot keep pace, and the body's Gln reservoir (mainly skeletal muscle) becomes depleted rapidly.

**Figure 1:**
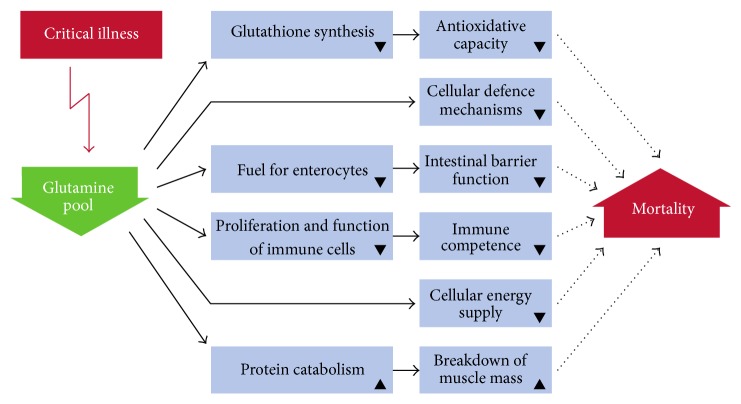
Consequences of glutamine depletion for the organism (modified from Wischmeyer 2003 [45]).

Gln depletion is noticeable by marked reductions of Gln in muscle, immunologic tissues, and (in many cases) blood, with consequences such as reduced protein synthesis, muscle loss, and physical fatigue [40, 45, 46]. These metabolic impairments limit the function of key tissues and cells, in particular that of the immune system, as well as the cellular mechanisms of stress response (Figure 1) [45, 47, 48].

It can be concluded that the consumption of Gln during metabolic stress is generally higher than Gln endogenous synthesis, and so the body lacks Gln to support essential pathways promoting healing processes. Thus, exogenous Gln supply is an important countermeasure to avoid the adverse consequences of Gln deficiency which may result in an increased risk of sepsis, MOF, and premature death [45]. Consequently, Gln has to be classified as a conditionally indispensable nutrient in critical illness and must be supplied exogenously with any nutritional regimen [49, 50].

## 3. Parenteral Gln Nutrition in Critical Illness: Clinical Trials

Whilst Gln is a “natural” component of proteins added to enteral formulations, standard amino acid solutions for PN are lacking this amino acid. The major limitations preventing the addition of free Gln to parenteral amino acid preparations are (a) the poor solubility of Gln in water and (b) the generation of potentially toxic pyroglutamic acid from free Gln during heat sterilization and storage. To solve these problems Gln-containing dipeptides, L-alanyl-L-glutamine (Ala-Gln) and glycyl-L-glutamine (Gly-Gln), were developed. These alternative Gln sources for PN are stable and soluble. As Gln is bound at the N-terminal position of these dipeptides, the *α*-amino group is protected and the dipeptides can safely be included in or added to any parenteral amino acid solution or ready-to-use multichamber bag [9, 51]. Ala-Gln and Gly-Gln are rapidly hydrolyzed following intravenous injection, resulting in the almost instantaneous release of the constituent amino acids [52–56]. Consequently, in most of the randomized controlled clinical trials (RCTs) performed, Gln peptides were used as efficient Gln sources.

### 3.1. Clinical Nutrition Studies

Benefits of parenteral Gln dipeptides in critical care have been continuously and repeatedly demonstrated in numerous RCTs. Compared with Gln-free PN, Gln-supplemented PN improved nitrogen economy, normalized IGF-I levels [57, 58], increased serum HSP-70 levels [59], decreased gut permeability and endotoxin levels [60], decreased infectious morbidity [61–66], improved glycemic control [61, 62, 67], exerted immunomodulatory effects, decreased APACHE-II score and MODS [68], decreased wound healing time, and lowered the cost of hospitalization [60]. In patients with severe acute pancreatitis, complication rates and mortality were reduced and length of stay (LOS) was shortened [66, 69].

For example, Déchelotte et al. [61] included 114 patients from 16 ICUs in France, admitted for multiple trauma, complicated surgery, or pancreatitis. Ala-Gln-supplemented total PN (TPN) (0.5 g/kg BW/day, corresponding to 0.3 g Gln/kg BW/day) was associated with a significantly lower incidence of complicated outcome, mainly owing to fewer infections. Moreover, the incidence of hyperglycemia and the number of patients needing insulin were lower in the Ala-Gln group. Grau et al. [62] conducted a multicentre RCT in 12 Spanish ICUs including 127 patients with APACHE II scores greater than 12 and requiring PN for 5–9 days. The group of patients receiving total PN (TPN) with Ala-Gln (0.5 g/kg BW/day) had a significantly reduced incidence of nosocomial pneumonia and urinary tract infections. Moreover, insulin dose and insulin resistance were significantly reduced in the Ala-Gln-supplemented group, indicative of improved glycemic control. In surgical ICU patients (excluding those undergoing pancreatic surgery), Estívariz et al. [64] were able to demonstrate that Ala-Gln-supplemented PN (0.5 g/kg BW/day) was associated with significantly decreased total nosocomial infections, bloodstream infections, pneumonia, and bacterial and fungal infections. Hospital mortality decreased by 35%, but results for this outcome narrowly failed to reach statistical significance.

These beneficial effects of parenteral Gln, proven in single-centre and larger multicentre studies, have been confirmed by recent meta-analyses [12, 13, 70]. In intensive care medicine it is well known that the heterogeneity of the patient population frequently impairs demonstration of treatment effects. Thus, whilst single-centre trials are usually underpowered for subgroup analyses, multicentre trials increase power with more patients but then between-centre heterogeneity can become more of an issue. Meta-analyses have thus proven to be extremely valuable tools in evidence-based medicine. They give greater precision and clinical applicability of results than individual studies [71, 72]. It is, thus, of utmost importance that two recent meta-analyses and a Cochrane database systematic review have confirmed the safety and efficacy of Gln-dipeptide supplemented PN [12, 13, 70].

Bollhalder et al., 2013 [13], conducted a meta-analysis including 40 RCTs in 3107 patients evaluating the effect of parenteral Gln supplementation. When only trials evaluating higher doses (>0.2 g/kg BW/day supplemental Gln, in accordance with current evidence-based recommendations such as those by ESPEN [11]) were studied, significant reductions in short-term mortality (*p* = 0.003), infections (*p* = 0.006), and hospital LOS (*p* = 0.001) were revealed across all patient populations included (surgery, critical illness, and mixed). Similarly, the meta-analysis by Wischmeyer et al., 2014 [12], included 26 well-designed trials published between 1997 and 2012 involving 2484 patients, demonstrating significant reductions of hospital mortality and LOS. Tao et al. [70] included RCTs published up to May 2013 in their 2014 Cochrane database review and found evidence that Gln supplementation in critically ill and/or surgical patients reduced infection rate, number of days on mechanical ventilation, and hospital LOS. A subgroup analysis of studies with intravenous Gln also revealed a significant reduction in short-term mortality within hospital stay or within 1 month [70].

Beyond its most important clinical benefits in the ICU, Gln supplementation of PN is also economically attractive. An economic evaluation revealed that a PN regimen supplemented with Ala-Gln is more cost-effective than standard PN regimens. Incorporating data from 200 Italian ICUs in over 60000 patients, Pradelli et al. demonstrated reductions in mortality rate, infection rate, and hospital LOS with Ala-Gln. This resulted in a lower total cost per patient and treatment cost was completely compensated by savings on ICU and antibiotic costs [73]. A recently published update of this evaluation [74] showed mean cost savings of €4991 per patient discharged alive or €1047 per patient admitted to the hospital using the same simulation model including efficacy data derived from the meta-analysis by Bollhalder et al. [13].

### 3.2. Gln as a High-Dose Supplement

In all the previously discussed trials, Gln was administered in-line with the clinical guidelines [12], which is exclusively via the parenteral route, using approved and recommended doses (0.3–0.5 g/kg BW/day), in combination with adequate nutrition to patients who were hemodynamically and metabolically stabilized, and excluding patients with hepatic and/or renal failure. In contrast, the REDOXS^©^ study [16–18] used a new approach, investigating Gln supplementation in a completely different way: not using Gln as a nutrient but rather as a component of a pharmaconutrition regimen and including high-risk patients presenting with more than two organ failures or shock [75]. Hence, REDOXS^©^ was designed as an exploratory study in which Gln was given as a combination of enteral (30 g/day from Ala-Gln + Gly-Gln) and intravenous (0.35 g/kg BW/day from Ala-Gln) supplements, resulting in an unphysiologically high Gln dose. Moreover, Gln was provided together with antioxidants and was dissociated from adequate nutritional support. More than 30% of patients presented with renal failure and more than 90% with shock at admission, both of which are long-standing contraindications for Gln supplementation.

Indeed, the scientifically sound and accepted background to administer Gln to severely ill patients is to optimize nutritional support by including an obviously conditionally indispensable substrate [76]. All the beneficial aspects of Gln provision can be explained by its central role in various metabolic pathways. An adequate nutritional support always requires a balanced provision of energy and all indispensable nutrients; to give high doses of one nutrient without supplying sufficient energy, nitrogen and macro- and micronutrients cannot be successful. It is, thus, not appropriate to consider findings from these “nonnutritive” exploratory studies (e.g., the REDOXS^©^ results), when formulating recommendations for parenteral nutrition.

## 4. Translation of Scientific Evidence into Clinical Practice

### 4.1. Select the Patients Benefiting from Gln-Containing Nutrition

All patients are at high risk of developing a negative Gln balance if they have undergone major surgery or trauma or are suffering from severe illness such as infection or organ failure, where vital functions need to be supported in the ICU by mechanical and/or pharmaceutical means for more than 72 hours (“critically ill patients”). If adequate nutritional support by the enteral route is not feasible, patients should receive total or supplemental PN providing sufficient energy, indispensable amino acids (routinely including Gln), and essential micronutrients [10, 11, 77]. Otherwise, protein-energy malnutrition will further aggravate the clinical situation and lessen the effectiveness of medical therapy.

Nearly all “positive” Gln studies excluded patients with severe liver and/or renal failure, or unresuscitated shock. The kidneys are central organs in Gln metabolism [78], implying that a loss of the kidneys' functional capacity—such as in patients with acute kidney injury (AKI) not receiving renal replacement therapy (RRT)—compromises the metabolic handling of supplemental Gln. Gln is quantitatively the most important donor of ammonia in the kidneys, which depends on Gln metabolism for acid-base buffering [78]. It has also been shown that gluconeogenesis from Gln occurs primarily in the kidney [79]. In AKI, ammonia elimination is impaired and the accumulating ammonia is toxic to the kidneys. Moreover, oxygen delivery is reduced and, therefore, might be inadequate to allow for the use of Gln as a gluconeogenic precursor. Thus, it was not surprising that a retrospective subgroup analysis of renal patients from the REDOXS^©^ trial revealed that outcome in patients with renal dysfunction at study enrolment who were given Gln was particularly unfavourable as reflected by increased 28-day mortality (*p* = 0.028 Gln versus no Gln) compared with results in the overall study population. This effect was even more pronounced in patients who previously did not need dialysis, whilst Gln had a positive effect in patients with preserved renal function and in patients on RRT [18]. All these findings clearly indicate that amino acid/protein administration in patients with AKI might be stage-specific. Patients in the “injury phase” of AKI according to the* Kidney Disease: Improving Global Outcomes (KDIGO) Transplant Work Group* classification [80] and with a creatinine clearance less than 25 mL/minute not receiving RRT should thus be excluded from Gln supplementation.

Likewise, because the liver has a central role in ammonia detoxification [78], particular caution is required in critically ill patients with liver failure when giving parenteral amino acids in general and Gln in particular. Nitrogen is transported from peripheral tissues (principally from muscle and the lungs) to the central organs as Gln, with the liver being the central site for nitrogen metabolism in the body. The periportal cells of the liver are characterized by high levels of the enzyme glutaminase, and here Gln metabolism provides ammonia for urea biosynthesis which is transported via the bloodstream to the kidney for elimination [78] whilst the Gln-carbon skeleton can serve as a substrate for gluconeogenesis [79]. In acute liver failure (ALF), ammonia metabolism in the periportal cells of the liver is severely impaired. Thus, ammonia is released in large amounts into the bloodstream, resulting in high blood ammonia levels. When considering Gln supplementation it is very important to differentiate between the different types of hepatic dysfunction. The early phase of fulminant ALF with hemorrhage and shock is a general contraindication to any form of nutrition, including Gln, but ALF developing from cirrhosis and acute-on-chronic liver failure does not generally represent a contraindication as long as plasma Gln and ammonia levels do not exceed the physiological range (<900 mmol/L).

It is also important to note that not all types of organ failure represent a contraindication against the administration of Ala-Gln. Whilst it is important to exclude patients with severe renal and/or hepatic insufficiency, Ala-Gln supplementation is generally safe in patients with brain damage, lung damage with adequate technical support, gastrointestinal failure with recompensated circulatory shock, and resolved metabolic acidosis as well as normal substrate utilization. Of course, whenever the clinical situation forbids clinical nutrition in general, Gln supplementation is also contraindicated.

### 4.2. Always Supply Parenteral Gln Peptides as Part of Proper Nutrition Support

In the REDOXS^©^ trial, beneficial effects could not be demonstrated when Gln was administered to patients who were, for the most part, inadequately nourished [16–18]. In a post hoc analysis it has recently been confirmed that the adequacy of clinical nutrition received in the ICU significantly influenced long-term outcomes in the REDOXS^©^ patients. The highest mortality risk was seen in patients receiving 50% or less of their caloric goals [81].

### 4.3. Use the Right Dose

Gln at a dose of up to 10 g/day, corresponding to the intake from a normal mixed diet, should be a mandatory part of any clinical nutrition regimen. In situations when this basic supply is insufficient, such as in critical illness, additional Gln should be given to provide sufficient Gln, up to a maximum total dose of 30 g/day. This Gln administration should be maintained throughout the hypercatabolic phase, signified by increased CRP, decreased prealbumin levels, and negative nitrogen balance. In accordance with present guidelines, supplemental Gln should be administered intravenously at a dose of 0.3–0.6 g Ala-Gln dipeptide/kg BW/day [11]; higher peptide doses given as separate supplements may result in metabolic imbalances.

## 5. Summary and Conclusions

Gln is conditionally indispensable in critical illness for the maintenance of intestinal integrity and function, support of the immune system, and maintenance of antioxidative balance through multiple mechanisms. Data from controlled clinical trials and meta-analyses have confirmed that adhering to the well-established use of intravenous Gln, in combination with adequate nutritional support, reduces mortality, infectious complications, and ICU/hospital LOS, whilst also being an economically attractive treatment. Suitable candidates for intravenous Gln include, in particular, critically ill patients with burns, trauma, or malignancies. Although Gln is contraindicated in patients with severe renal and/or hepatic insufficiency, it is generally safe in patients with failures of other organs (e.g., brain damage or respiratory failure receiving adequate mechanical ventilation or gastrointestinal failure) with recompensated circulatory shock and resolved metabolic acidosis as well as normal substrate utilization.
